# On the role of A_2A_ and D_2_ receptors in control of cocaine and food-seeking behaviors in rats

**DOI:** 10.1007/s00213-014-3818-5

**Published:** 2014-11-26

**Authors:** Karolina Wydra, Agata Suder, Dasiel O. Borroto-Escuela, Malgorzata Filip, Kjell Fuxe

**Affiliations:** 1 Laboratory of Drug Addiction Pharmacology, Department of Pharmacology, Institute of Pharmacology, Polish Academy of Sciences, Smętna 12, 31-343 Kraków, Poland; 2 Department of Neuroscience, Karolinska Institutet, Stockholm, Sweden

**Keywords:** Adenosine (A)_2A_ receptor ligands, Cocaine-seeking behavior, Food-seeking behavior, Quinpirole, Rat

## Abstract

Recent studies indicate that adenosine may influence dopamine neurotransmission via A_2A_ receptors which antagonistically interact with D_2_ receptor-mediated signaling in the brain. We examined the effects of selective A_2A_ receptor ligands such as the agonist CGS 21680 and the antagonists KW 6002 or SCH 58261 as well as of the D_2_-like receptor antagonist raclopride on reinstatement of cocaine seeking induced by cocaine, the cocaine-conditioned cue, or the D_2_-like receptor agonist quinpirole in rats. For comparison, effects of the A_2A_ receptor ligands on reinstatement of food seeking were also studied. CGS 21680 significantly attenuated the reinstatement of cocaine (ip) seeking, and even more potently it reduced quinpirole (ip) or the cue-induced relapse of cocaine seeking as well as cue-induced food seeking. A potent reduction toward the cocaine-, quinpirole-, or cue-induced reinstatement of cocaine seeking was seen with raclopride. Pretreatment with KW 6002 or SCH 58261 reinstated cocaine seeking, and such increases were blocked by raclopride. In the higher doses, KW 6002 or SCH 58261 evoked food-seeking. In combination with the subthreshold dose of cocaine (2.5 mg/kg) or with the cue, low doses of KW 6002 but not SCH 58261 reinstated cocaine-seeking behavior, while none of the A_2A_ receptor antagonists affected the cue-induced food-seeking behavior. The results indicate that A_2A_ activation and D_2_-like receptor blockade counteract cocaine and food relapse. It is proposed that A_2A_ receptor- and D_2_ receptor-mediated adenosine and dopamine signaling antagonistically interact in the striato-pallidal GABA neurons to regulate cocaine and food-seeking behavior.

## Introduction

Cocaine addiction is characterized by an inability of addicts to inhibit drug use relapse triggered by drugs, environmental cues, or stressful life events (Belin et al. 2013; Everitt and Heberlein 2013). Similar factors are active in a rat model of relapse where animals extinguished from cocaine self-administration reinstate seeking behavior following cocaine priming dose, the drug-paired conditioned stimulus, or stressors (Fuchs et al. 1998; Markou et al. 1993). The neuronal basis of cocaine relapse includes activation of the mesocorticolimbic circuitry with changes in glutamate and dopamine (DA) neurotransmissions (Kalivas 2009; Sinha 2013).

Recent data indicate that adenosine may influence DA and glutamatergic neurotransmission, especially in the striatal region (Fuxe et al. 2007a, 2008). As shown for adenosine and DA signaling in striato-pallidal γ-aminobutyric acid (GABA) neurons, D_2_ receptor-mediated DA transmission can be reduced by A_2A_ receptor agonists, while A_2A_ receptor antagonists increase it (Filip et al. 2012; Fuxe et al. 2007b, 2010). Stimulation of D_2_ receptors in the ventral and dorsal striatum is involved in mediating the locomotor, sensitizing, and rewarding effects of cocaine or other drugs of abuse, and these actions are antagonized by activation of A_2A_ receptors (Bachtell and Self 2009; Filip et al. 2006, 2012; Jastrzębska et al. 2014; Knapp et al. 2001).

The role of A_2A_ receptors in cocaine relapse in the frame of the antagonistic interactions in A_2A_ receptor and D_2_ receptor-mediated adenosine and DA signaling, respectively, has not been fully investigated so far. The aim of this study was to examine the role of A_2A_ receptors, using two selective antagonists (KW 6002 or SCH 58261) in cocaine-seeking behavior evoked by cocaine, the D_2_-like receptor agonist quinpirole, or the cue. The dependency on D_2_ receptors was tested with the D_2_-like antagonist raclopride. The two used A_2A_ receptor antagonists may possibly differentially influence mechanisms at pre- and postsynaptic sites in the rat striatum, as KW 6002 is the postsynaptic A_2A_ receptor antagonist while SCH 58261 is regarded as the mixed post- and presynaptic receptor antagonist (Filip et al. 2012). For comparison, effects of the A_2A_ receptor antagonists on reinstatement of food-seeking behavior were studied. To extend the existing knowledge on how A_2A_ receptor stimulation may oppose D_2_ receptor signaling in the rat ventral striatum to reduce cocaine relapse (Bachtell and Self 2009; O’Neill et al. 2012), we also examined the effects of the selective A_2A_ receptor agonist CGS 21680 on cocaine- and food-seeking behaviors.

## Experimental procedures

### Animals

Male Wistar rats (Charles River, Sulzfeld, Germany) weighing 280–300 g were used. The animals were housed individually in clear transparent home cages with free access to food (VRF1(p) pellets, UK) and water, at a room temperature of 20 ± 1 °C and at a 40–50 % humidity on a 12-h light–dark cycle (the light on at 6:00). During few days of initial lever press training performed in standard operant chamber (Med-Associates, USA), animals had limited access to water (rats used for cocaine self-administration) or food (rats used for food self-administration). Later on, rats used in cocaine self-administration procedures including extinction and reinstatement testing had free access to water and pellets, while those used in the food self-administration procedures including extinction and reinstatement testing had free access to water and limited pellet intake (20 g) after each session. On Saturdays, following experimental sessions, each rat used for food self-administration received 40 g of pellets. All the experiments were conducted during the light phase of the light–dark cycle (between 0700 and 1600 hours) and were carried out in accordance with the National Institutes of Health Guide for the Care and Use of Laboratory Animals and with the approval of the Animal Care and Use Committee of the Institute of Pharmacology, Polish Academy of Sciences in Krakow.

### Drugs

Cocaine hydrochloride (Sigma-Aldrich; USA), (E)-1,3-diethyl-8-(3,4-dimethoxystyryl)-7-methyl-3,7-dhydro-H-purine-2,6-dione hydrochloride, *istradefylline* (KW 6002; Tocris, UK), 7-(2-phenylethyl)-5-amino-2-(2-furyl)-pyrazolo-[4,3-e]-1,2,4-triazolo[1,5-c]pyrimidine hydrochloride (SCH 58261; Tocris, UK), 4-[2-[[6-amino-9-(*N*-ethyl-β-d-ribofuranuronamidosyl)-9*H*-purin-2-yl]amino]ethyl]benzene propanoic acid hydrochloride (CGS 21680; Tocris, UK) and trans-(e)-(4aR)-4,4a,5,6,7,8,8a,9-octahydro-5-propyl-1H-pyrazolo[3,4-g]quinoline monohydrochloride ((−)quinpirole; Sigma-Aldrich, USA), and (3,5-dichloro-N-[[(2S)-1-ethyl-2-pyrrolidinyl]methyl]-2-hydroxy-6-methoxy-benzamide) raclopride; Tocris, UK) were used. Cocaine, (−)quinpirole, CGS 21680, and raclopride were dissolved in 0.9 % NaCl; KW 6002 was dissolved in a mixture (1:1:8) of dimethyl sulfoxide (DMSO, Sigma-Aldrich, USA), Tween®80 (Sigma-Aldrich, USA), and 0.9 % NaCl, while SCH 58261 was dissolved in 1 % DMSO. (−)Quinpirole, CGS 21680, KW 6002, SCH 58261, and raclopride were injected ip 5, 10, 20, 30, and 40 min, respectively, before behavioral scoring in a volume of 0.1 ml/kg. Cocaine was administered iv in a volume of 0.1 ml per infusion or ip in a volume 0.1 ml/kg. Doses and pretreatment times of A_2A_ and D_2_ receptor ligands were established based on previous behavioral studies (Bachtell and Self 2009; Filip et al. 2006; Jastrzębska et al. 2014; Knapp et al. 2001; O’Neill et al. 2012; Wydra et al. 2014).

### Cocaine self-administration and extinction/reinstatement procedures

After 18-h water deprivation, animals were trained for 3 days to press a lever for 1 h daily in standard operant chambers (Med-Associates, USA) under a fixed ratio (FR) schedule 1 of water reinforcement. Two days after lever-pressing training and free access to food and water, the animals were implanted with a silastic®catheter in the external jugular vein, as described previously (Wydra et al. 2013). Catheters were flushed daily with 0.1 ml of a heparinized saline solution (70 U/ml, Biochemie, Austria) and 0.1 ml of a cephazolin solution (10 mg/ml Biochemie GmbH, Austria). Catheter patency was tested periodically or whenever an animal displayed behavior beyond baseline parameters using methohexital (10 mg/kg, iv), which induced the loss of consciousness within 5 s. No problems with catheter patency were reported in the tested rats. Rats were allowed 10 days to recover from surgery before the start of the experiments. Later on, all animals began lever pressing for cocaine reinforcement during 2-h daily sessions performed 6 days/week. Each completion of a FR 1 schedule on the “active” lever resulted in a 5-s injection of cocaine (0.5 mg/kg per infusion) together with a presentation of conditioned stimulus (light + tone). Following each injection, there was a 20-s time-out period during which responding was recorded but had no programmed consequences. Response on the “inactive” lever never resulted in cocaine delivery. Acquisition of the conditioned operant response lasted a minimum 9 days until subjects met the following criteria: minimum requirement of 24 reinforcements and active lever presses with average of six consecutive days varied by <10 % (Wydra et al. 2013). Next, the extinction procedure was carried out during which rats had 2-h daily training sessions with no cocaine delivery or presentation of the conditioned stimulus. Once they reached the extinction criteria (a minimum of ten extinction days with the responding to the active lever below 10 % of the level observed during the three consecutive days of maintenance. Separate groups of animals were tested for response reinstatement induced by a non-contingent presentation of cocaine (2.5 and 10 mg/kg, ip), a conditioned cue (tone + light previously paired with cocaine self-administration), quinpirole (0.125–0.5 mg/kg, ip), or A_2A_ receptor antagonists alone.

The effects of raclopride (0.1–0.4 mg/kg, ip) were tested on cocaine (10 mg/kg, ip), conditioned cue, quinpirole (0.5 mg/kg, ip), KW6002, or SCH 58261 induced cocaine seeking. Furthermore, the effect of a subthreshold dose of the receptor A_2A_ antagonists with cocaine (2.5 mg/kg, ip) or cue was investigated on cocaine seeking. During the reinstatement tests (2-h sessions), active lever presses on the FR 1 schedule resulted in intravenous injection of saline only. Drug combination was given in a randomized order in maximum of three reinstatement tests. Each rat underwent only one type of the reinstatement procedure (cocaine, cue, or A_2A_ and D_2_ receptor ligands) in maximally three reinstatement tests. The order of injections was counterbalanced according to a Latin square design, and the test sessions were separated by at least two to three baseline days of the extinction sessions.

### Food self-administration and extinction/reinstatement procedures

Food self-administration was conducted in a similar manner to cocaine self-administration, as described previously (Wydra et al. 2013). Food (pellet)-restricted rats (20 g/rat/day) were trained to press the lever in standard operant chambers (Med-Associates, St. Albans, GA, USA) under a FR 1 schedule of reinforcement (each completion of a FR 1 schedule on the “active” lever resulted in a delivery of the portion of sweetened milk (0.1 ml)) in daily 2-h sessions. Response on the “inactive” lever never resulted in sweetened milk delivery. Training and maintenance sessions occurred over a total of 14–16 days during which subjects met acquisition criteria that required the number of reinforcements and active lever presses over six consecutive maintenance sessions to vary by only 10 %. Once stable rates of responding were established, the extinction procedure started on the following day. During extinction sessions, subjects had 2-h daily training sessions with no delivery of sweetened milk. Once they reached the extinction criteria (a minimum of ten extinction days with the responding on the active lever below 10 % of the level observed during maintenance during at least three consecutive days), rats were divided into separate groups to run reinstatement tests evoked by either a presentation of sweetened milk or a cue (tone) previously paired with sweetened milk delivery. During the cue reinstatement tests (2-h sessions), active lever presses on the FR 1 schedule did not result in sweetened milk delivery. Separate groups of animals were tested for response reinstatement induced by a contingent presentation of sweetened milk, or a conditioned cue (tone previously paired with self-administered sweetened milk), or by A_2A_ receptor antagonists alone. Each rat underwent only one type of the reinstatement procedure (sweetened milk, cue, or A_2A_ receptor antagonists) in maximally three reinstatement tests. The order of injections was counterbalanced according to a Latin square design, and the test sessions were separated by at least two to three baseline days of the extinction sessions.

### Statistical analyses

The number of responses on the active and inactive levers and the number of sweetened milk reinforcements are shown as mean (±SEM). The effects of cocaine, cue, A_2A_, and D_2_ receptor ligands during reinstatement tests on the lever presses were analyzed using the two-way ANOVA for factors treatment, lever, and treatment × lever interaction or with three-way ANOVA for factors pretreatment, treatment, lever, and pretreatment × treatment × lever interaction. Post-hoc analyses for significant effects were performed with the Newman–Keuls’ test. A one-way ANOVA was used to analyze the number of sweetened milk reinstatement followed by the post-hoc Dunnett’s test to show differences between group means. A priori comparison with Student’s *t* test was included to analyze the effect of food reinstatement. The criterion for statistically significant differences was *p* < 0.05.

## Results

### Reinstatement of cocaine-seeking behavior

After about nine experimental sessions, rats met the criterion of a stable cocaine (0.5 mg/kg/infusion) self-administration. During maintenance phase, the mean numbers of responses emitted on active lever ranged from 38 to 46, while the number of inactive lever presses did not exceed 7. The daily mean cocaine intake amounts to 12.5–15 mg/kg/day. Neither lever responding nor cocaine intake varied between groups of rats.

#### Effects of cocaine, quinpirole, or cue

**Fig. 1:**
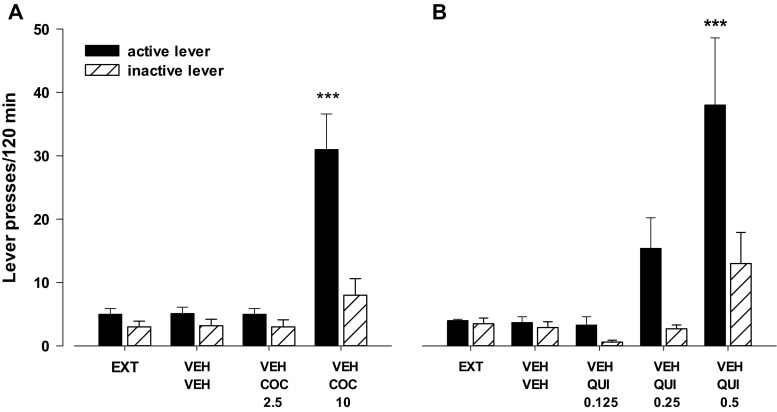
Effects of cocaine (COC; **a**) and quinpirole (QUI; **b**) on the reinstatement of cocaine-seeking behaviors in rats extinguished from cocaine (0.5 mg/kg) self-administration. The number of active and inactive lever presses is shown as mean (±SEM) for six to eight rats/group. ^***^ *p* < 0.001 vs. vehicle (VEH) + vehicle (Newman-Keuls test)

**Fig. 2:**
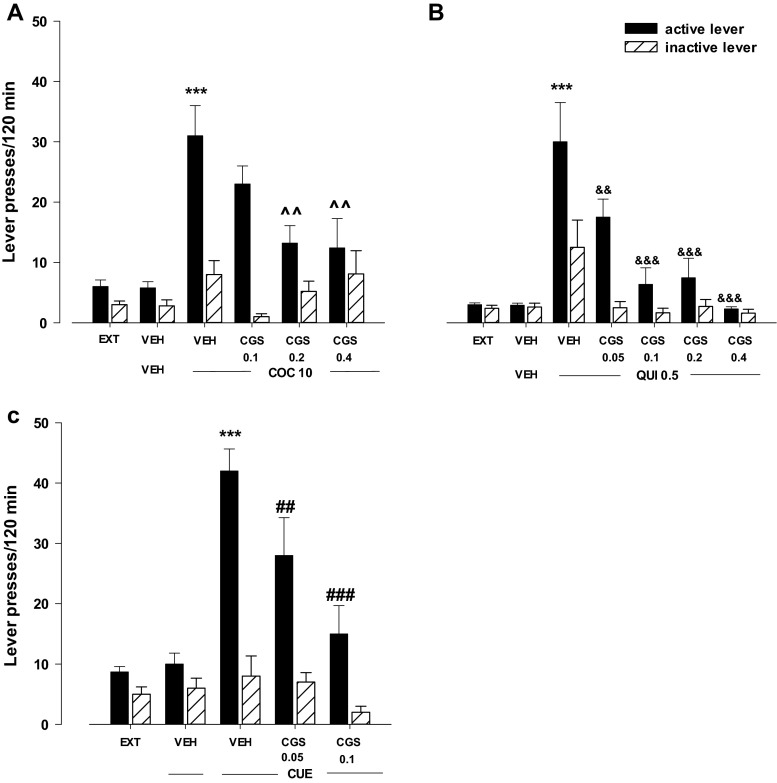
Effects of the A_2A_ receptor agonist CGS 21680 (CGS) on cocaine- (COC, 10 mg/kg; **a**), quinpirole- (QUI, 0.5 mg/kg; **b**), or cue- (light and tone previously associated with cocaine self-administration; **c**) induced reinstatement of cocaine-seeking behaviors in rats extinguished from cocaine (0.5 mg/kg) self-administration. The number of active and inactive lever presses is shown as mean (±SEM) for six to eight rats/group. ^***^ *p* < 0.001 vs. vehicle (VEH) + VEH; ^^^*p* < 0.01 vs. VEH + COC; &&*p* < 0.01, &&&*p* < 0.001 vs. VEH + QUI 0.5; ^##^ *p* < 0.01, ^###^ *p* < 0.001 vs. VEH + CUE (Newman-Keuls test)

After 10 days of extinction trials, the rats were tested for the response reinstatement induced by cocaine (2.5 or 10 mg/kg, ip; Fig. 1a), or quinpirole (0.125, 0.25, or 0.5 mg/kg, ip; Fig. 1b), or presentation of the cue paired earlier with cocaine infusions (Fig. 2c). The two-way ANOVA (treatment × lever interaction) indicated a significant effect of cocaine (*F*_2.36_ = 10.61, *p* < 0.01), quinpirole (*F*_3.48_ = 2.95, *p* < 0.05), and cue (*F*_1.23_ = 24.29, *p* < 0.001) on cocaine-seeking behavior.

The post-hoc Newman-Keuls test revealed that cocaine (10 mg/kg), quinpirole (0.5 mg/kg), and the cue significantly enhanced responding on the active lever (*p* < 0.001) without any changes in the number of inactive lever presses.

#### Effects of the A_2A_ receptor agonist CGS 21680 on cocaine-, quinpirole-, or cue-induced reinstatement

The two-way ANOVA for treatment × lever interaction indicated a significant effect of CGS21680 (0.1–0.4 mg/kg) on cocaine (10 mg/kg)-induced reinstatement (*F*_3.48_ = 3.86, *p* < 0.05). The post-hoc Newman-Keuls test revealed that CGS 21680 in doses of 0.2 and 0.4 mg/kg reduced the number of active lever presses (*p* < 0.01) without any changes in the number of inactive lever presses (Fig. 2a).

The two-way ANOVA for treatment × lever interaction indicated a significant effect of CGS21680 (0.05–0.4 mg/kg) on quinpirole (0.5 mg/kg)-induced reinstatement (*F*_3.48_ = 2.95, *p* < 0.05). The post-hoc Newman-Keuls test revealed that CGS 21680 dose-dependently and significantly (*p* < 0.01 for the lowest dose, and *p* < 0.001 for the higher doses) reduced the number of active lever presses without any changes in the number of inactive lever presses (Fig. 2b).

The two-way ANOVA for treatment × lever interaction indicated a significant effect of CGS 21680 (0.05–0.1 mg/kg) on cue-induced reinstatement (*F*_2.34_ = 3.83, *p* < 0.05). The post-hoc Newman-Keuls test revealed that CGS 21680 in doses of 0.05 and 0.1 mg/kg reduced the number of active lever presses (*p* < 0.01 and *p* < 0.001, respectively) without any changes in the number of inactive lever presses (Fig. 2c).

#### Effects of the D_2_-like receptor antagonist raclopride on cocaine-, quinpirole-, or cue-induced reinstatement

**Fig. 3:**
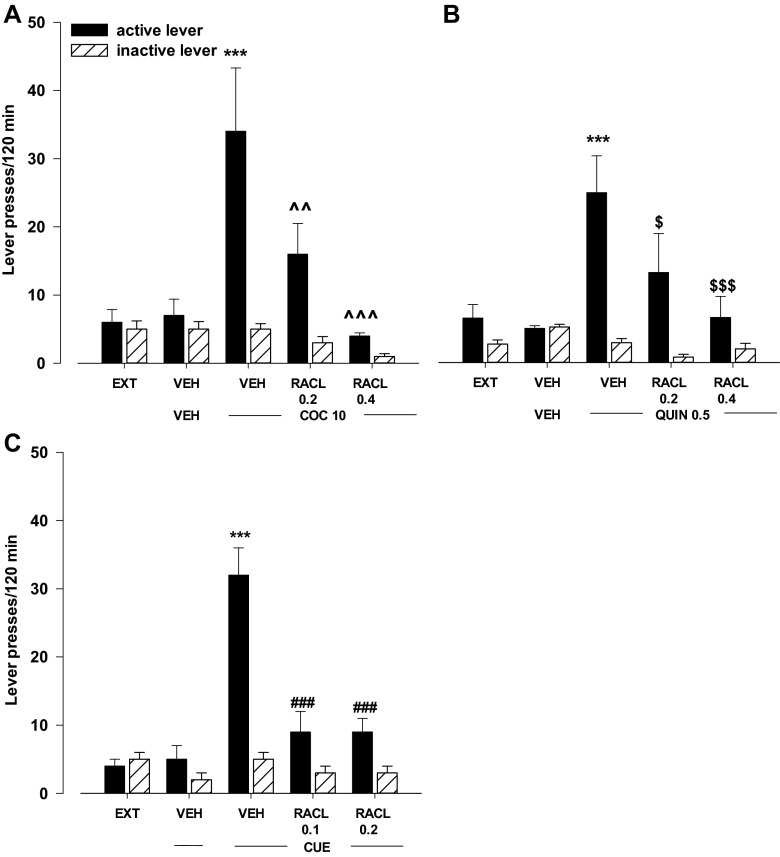
Effects of the D_2_-like receptor antagonist raclopride (RACL) on cocaine- (COC, 10 mg/kg; **a**), quinpirole- (QUIN, 0.5 mg/kg; **b**), or cue- (light and tone previously associated with cocaine self-administration; **c**) induced reinstatement of cocaine-seeking behaviors in rats extinguished from cocaine (0.5 mg/kg) self-administration. The number of active and inactive lever presses is shown as mean (±SEM) for five to seven rats/group. ^***^ *p* < 0.001 vs. vehicle (VEH) + VEH and VEH; ^^*p* < 0.01, ^^^*p* < 0.001 vs. VEH + COC; ^$^ *p* < 0.05, ^$$$^ *p* < 0.001 vs. VEH + QUI 0.5; ^###^ *p* < 0.001 vs. VEH + CUE (Newman-Keuls test)

The two-way ANOVA for treatment × lever interaction indicated a significant effect of raclopride (0.2–0.4 mg/kg) on cocaine-induced reinstatement (*F*_2.30_ = 5.00, *p* < 0.01). The post-hoc Newman-Keuls test revealed that raclopride in doses of 0.2 and 0.4 mg/kg significantly (*p* < 0.01 at the low dose, and *p* < 0.001 at the high dose) reduced the number of active lever presses without any changes in the number of inactive lever presses (Fig. 3a).

The two-way ANOVA for treatment × lever interaction indicated a significant effect of raclopride (0.2–0.4 mg/kg) on quinpirole-induced reinforcement (*F*_2.28_ = 3.87, *p* = 0.03). The post-hoc Newman-Keuls test revealed that raclopride in doses of 0.2 and 0.4 mg/kg significantly (*p* < 0.05 at the low dose, and *p* < 0.001 at the high dose) reduced the number of active lever presses without any changes in the number of inactive lever presses (Fig. 3b).

The two-way ANOVA for treatment × lever interaction indicated a significant effect of raclopride (0.1–0.2 mg/kg) on the cue-induced reinstatement (*F*_2.32_ = 15.03, *p* < 0.001). The post-hoc Newman-Keuls test revealed that raclopride in both doses, 0.1 and 0.2 mg/kg, highly significantly (*p* < 0.001) reduced the number of active lever presses without any changes in the number of inactive lever presses (Fig. 3c).

#### Effects of the A_2A_ receptor antagonists KW 6002 and SCH 58261

**Fig. 4:**
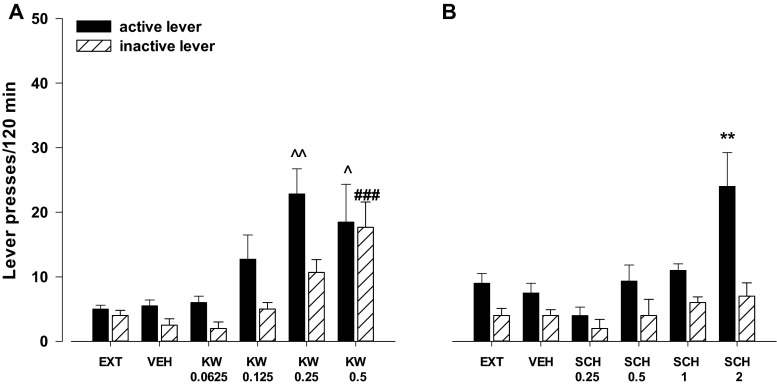
Effects of the A_2A_ receptor antagonists KW 6002 (KW, 0.0625–0.5 mg/kg; **a**) and SCH 58261 (SCH, 0.25–2 mg/kg; **b**) on the reinstatement of cocaine-seeking behaviors in rats extinguished from cocaine (0.5 mg/kg) self-administration. The number of active and inactive lever presses is shown as mean (± SEM) for six to eight rats/group. ^**^ *p* < 0.01 vs. vehicle (VEH) (Newman-Keuls test); ^*p* < 0.05, ^^*p* < 0.01 vs. VEH (active lever), ^###^ *p* < 0.001 vs. VEH (inactive lever) (Dunnett’s test)

The two-way ANOVA for treatment × lever interaction did not indicate a significant effect of KW 6002 (0.0625–0.5 mg/kg) on cocaine-seeking behavior (*F*_4.52_ = 1.50, *p* = 0.21). However, the one-way ANOVA analysis showed a significant effect of KW 6002 on active (*F*_4.25_ = 4.96, *p* < 0.01) and inactive (*F*_4.25_ = 6.68, *p* < 0.001) levers. The post-hoc Dunnett’s test revealed that KW 6002 in doses of 0.25 and 0.5 mg/kg significantly (*p* < 0.01 and *p* < 0.05, respectively) increased the number of active lever presses. Additionally, KW 6002 in a dose of 0.5 mg/kg increased (*p* < 0.001) the number of inactive lever presses (Fig. 4a).

The two-way ANOVA for treatment × lever interaction indicated a significant effect of SCH 58261 (0.25–2 mg/kg) on cocaine-seeking behavior (*F*_4.50_ = 1.51, *p* = 0.05). The post-hoc Newman-Keuls test revealed that SCH 58261 in a dose of 2 mg/kg increased the number of active lever presses (*p* < 0.01) without any changes in the number of inactive lever presses (Fig. 4b).

#### Effects of the A_2A_ receptor antagonists KW 6002 and SCH 58261 on cocaine- or cue-induced reinstatement

**Fig. 5:**
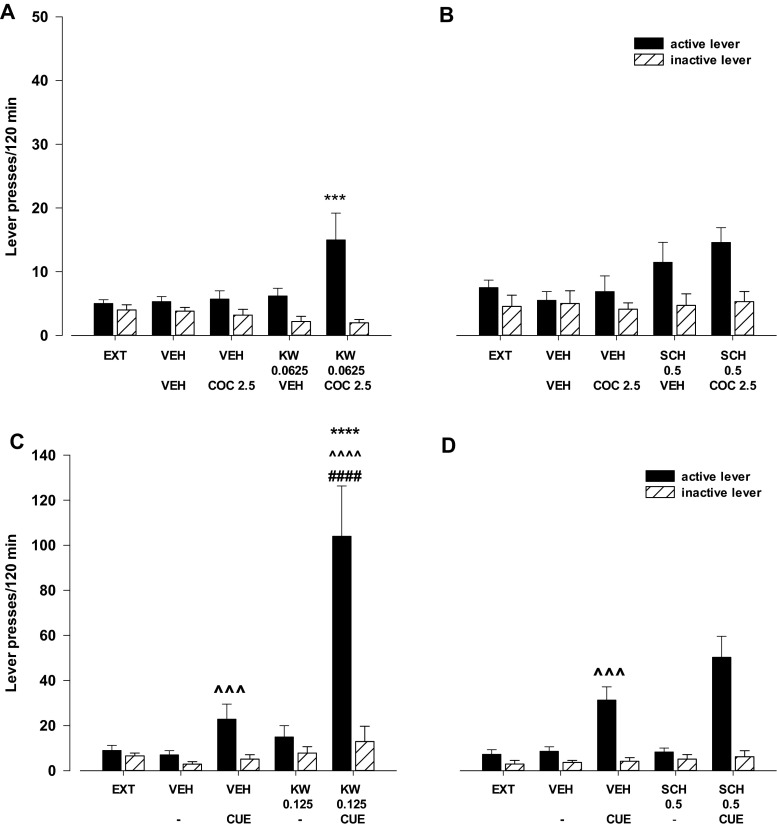
Effects of the A_2A_ receptor antagonists KW 6002 (KW) and SCH 58261 (SCH) on cocaine- (COC, 2.5 mg/kg; **a**, **b**) or cue- (light and tone previously associated with cocaine self-administration; **c**, **d**) induced reinstatement of cocaine-seeking behaviors in rats extinguished from cocaine (0.5 mg/kg) self-administration. The number of active and inactive lever presses is shown as mean (±SEM) for six to eight rats/group. ^***^ *p* < 0.001 vs. vehicle (VEH) + VEH, ^^^^^ *p* < 0.001, ^^^^^^ *p* < 0.0001 vs. VEH + (−); ^aaaa^ *p* < 0.0001; vs. VEH + CUE; ^####^ *p* < 0.0001 vs. VEH + KW 0.125 (Newman-Keuls test)

The three-way ANOVA for pretreatment × treatment × lever interaction indicated a significant effect of KW 6002 (0.0625 mg/kg) on a subthreshold dose of cocaine (2.5 mg/kg) for reinstatement (*F*_1.46_ = 4.343, *p* < 0.05). The post-hoc Newman-Keuls test revealed that KW 6002 in a dose of 0.0625 mg/kg in combination with cocaine (2.5 m/kg) increased the number of active lever presses (*p* < 0.001) without any changes on the number of inactive lever presses (Fig. 5a).

The three-way ANOVA for pretreatment × treatment × lever interaction indicated a nonsignificant effect of SCH 58261 (0.5 mg/kg) on a subthreshold dose of cocaine (2.5 mg/kg) for reinstatement (*F*_1.54_ = 0.179; *p* = 0.67) (Fig. 5b).

The three-way ANOVA for pretreatment × treatment × lever interaction indicated a significant effect of KW 6002 (0.125 mg/kg) on cue-induced reinstatement (*F*_1.48_ = 5.43, *p* < 0.05). The post-hoc Newman-Keuls test revealed that KW 6002 in a dose of 0.125 mg/kg in combination with the cue increased the number of active lever presses (*p* < 0.001) without any changes in the number of inactive lever presses (Fig. 5c).

The three-way ANOVA for pretreatment × treatment × lever interaction indicated a nonsignificant effect of SCH 58261 (0.5 mg/kg) on cue-induced reinstatement (*F*_1.40_ = 2.24, *p* = 0.142), while the two-way ANOVA for treatment × lever interaction indicated a significant effect (*F*_1.20_ = 36.20, *p* < 0.001; Fig. 5d). However, the post-hoc Newman-Keuls test did not reveal any effect of SCH 58261 in combination with the cue.

#### Effects of the D_2_-like receptor antagonist raclopride on KW 6002- or SCH 58261-induced reinstatement

**Fig. 6:**
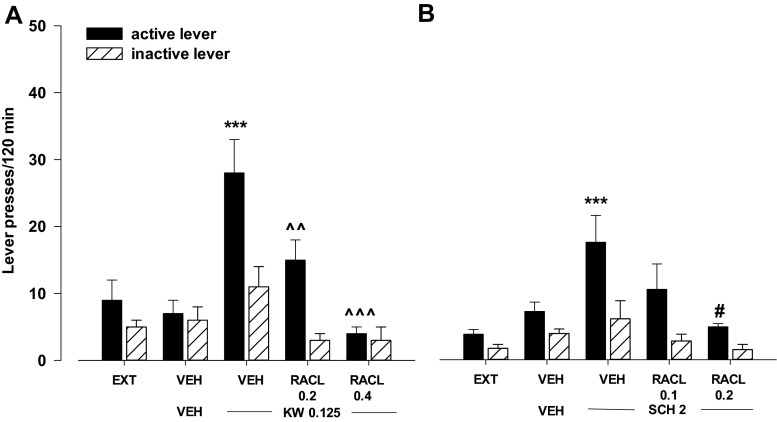
Effects of the D_2_-like receptor antagonist raclopride (RACL) on KW 6002- (KW, 0.125 mg/kg; **a**) and SCH 58261- (SCH, 2 mg/kg; **b**) induced reinstatement of cocaine-seeking behaviors in rats extinguished from cocaine (0.5 mg/kg) self-administration. The number of active and inactive lever presses is shown as mean (±SEM) for six rats/group. ^***^ *p* < 0.001 vs. vehicle (VEH) + VEH (Newman-Keuls test); ^^*p* < 0.01, ^^^*p* < 0.001 vs. VEH + KW 0.125 (Newman-Keuls test), ^#^ *p* < 0.05 vs. VEH + SCH 2 (Dunnett’s test)

The two-way ANOVA for treatment × lever interaction indicated a significant effect of raclopride (0.2–0.4 mg/kg) on KW 6002-induced reinstatement for active lever presses (*F*_2.24_ = 4.75, *p* < 0.01). The post-hoc Newman-Keuls test revealed that raclopride in doses of 0.2 and 0.4 mg/kg significantly (*p* < 0.01 at the low dose, and *p* < 0.001 at the high dose) reduced the number of active lever presses without any changes in the number of inactive lever presses (Fig. 6a).

The two-way ANOVA for treatment × lever interaction did not indicate a significant effect for raclopride (0.1–0.2 mg/kg) on SCH 58261-induced reinstatement (*F*_2.30_ = 2.19, *p* = 0.13), while the one-way ANOVA showed reduced number of active lever presses at 0.2 mg/kg of raclopride (*F*_2.15_ = 4.26, *p* < 0.05) without any changes in the number of inactive lever presses (*F*_2.15_ = 2.23, *p* = 0.14; Fig. 6b).

### Reinstatement of food-seeking behavior

After about 14 experimental sessions rats met the criterion of a stable food (sweetened milk) self-administration. During maintenance phase, the mean numbers of responses emitted on active lever ranged from 1,300 to 1,400, while the number of inactive lever presses did not exceed 30. The daily mean sweetened milk intake ranged from 240 to 250 portions. Neither lever responding nor sweetened milk intake varied between groups of rats.

#### Effects of food or cue

**Fig. 7:**
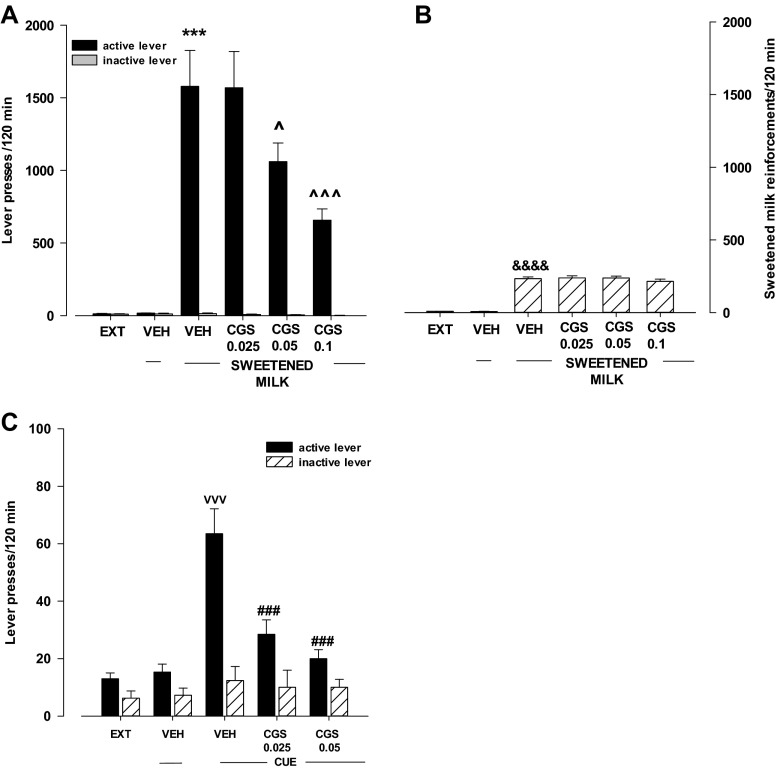
Effects of the A_2A_ receptor agonist CGS 21680 (CGS) on food (sweetened milk)- (**a**, **b**) or cue- (**c**) induced reinstatement of food seeking behavior in rats extinguished from food self-administration The number of active and inactive lever presses and the number of food reinforcements is shown as mean (±SEM) for seven to eight rats/group. ^***^ *p* < 0.001 vs. vehicle (VEH); ^*p* < 0.05; ^^^*p* < 0.001 vs. VEH + FOOD; ^VVV^ *p* < 0.001 vs. VEH; ^###^ *p* < 0.001 vs. VEH + CUE (Newman-Keuls test). &&&&*p* < 0.0001 vs. VEH (Student’s *t* test)

After 10 days of extinction trials, the rats were tested for the response reinstatement induced by sweetened milk (Fig. 7a, b) or presentation of the cue paired earlier with sweetened milk delivery (Fig. 7c). Both reinstatement of sweetened milk delivery and the cue significantly enhanced responding on the active lever (*p* < 0.001) without any changes in the number of inactive lever presses.

#### Effects of the A_2A_ receptor agonist CGS 21680 on food- or cue-induced reinstatement

The two-way ANOVA for treatment × lever interaction indicated a significant effect of CGS21680 (0.025–0.1 mg/kg) on sweetened milk-induced reinstatement for lever presses (*F*_3.56_ = 5.36, *p* < 0.01). The post-hoc Newman-Keuls test revealed that CGS 21680 in doses of 0.05 and 0.1 mg/kg significantly (*p* < 0.05 at the low dose, *p* < 0.001 at a high dose) reduced the number of active lever presses, without any changes in the number of inactive lever presses (Fig. 7a).

The one-way ANOVA did not indicate a significant effect of CGS 21680 (0.025–0.1 mg/kg) on sweetened milk reinforcement (*F*_3.28_ = 0.624, *p* = 0.61; Fig. 7b). However, a priori comparison with Student’s *t* test indicated a significant effect of sweetened milk reinstatement (dt = −10.87, *p* < 0.00001; Fig. 7b).

The two-way ANOVA for treatment × lever interaction indicated a significant effect of CGS21680 (0.025–0.05 mg/kg) on the cue-induced reinstatement (*F*_2.42_ = 10.57, *p* < 0.001). The post-hoc Newman-Keuls test revealed that CGS 21680 in both doses, 0.025 and 0.05 mg/kg, significantly (*p* < 0.001) reduced the number of active lever presses, without any changes in the number of inactive lever presses (Fig. 7c).

#### Effects of the A_2A_ receptor antagonists KW 6002 and SCH 58261

**Fig. 8:**
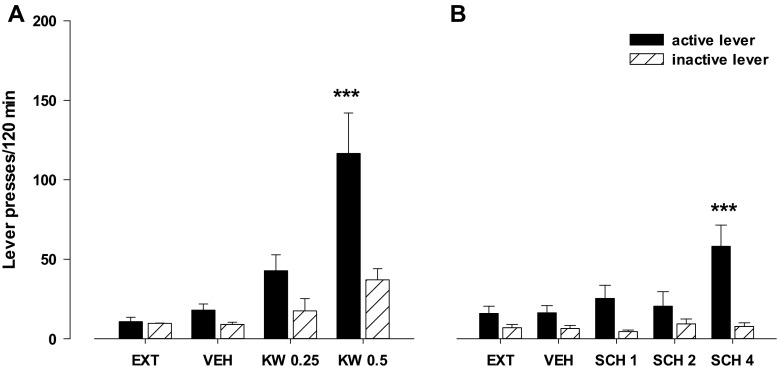
Effects of the A_2A_ receptor antagonists KW 6002 (KW, 0.25–0.5 mg/kg; **a**) and SCH 58261 (SCH, 1–4 mg/kg; **b**) on the reinstatement of food-seeking behaviors in rats extinguished from food self-administration. The number of active and inactive lever presses is shown as mean (±SEM) for eight rats/group. ^***^ *p* < 0.001 vs. vehicle (VEH) (Newman-Keuls test)

The two-way ANOVA for treatment × lever interaction indicated a significant effect of KW 6002 (0.025–0.5 mg/kg) on food (sweetened milk)-seeking behavior (*F*_2.42_ = 6.86, *p* < 0.01). The post-hoc Newman-Keuls test revealed that KW 6002 in a dose of 0.5 mg/kg increased the number of active lever presses (*p* < 0.001) without significant changes in the number of inactive lever presses (Fig. 8a).

The two-way ANOVA for treatment × lever interaction indicated a significant effect of SCH 58261 (1–4 mg/kg) on food (sweetened milk)-seeking behavior (*F*_3.56_ = 3.84, *p* < 0.01). The post-hoc Newman-Keuls test revealed that SCH 58261 in a dose of 4 mg/kg significantly increased the number of active lever presses (*p* < 0.001) without changes in the number of inactive lever presses (Fig. 8b).

#### Effects of the A_2A_ receptor antagonists KW 6002 and SCH 58261 on cue-induced reinstatement

**Fig. 9:**
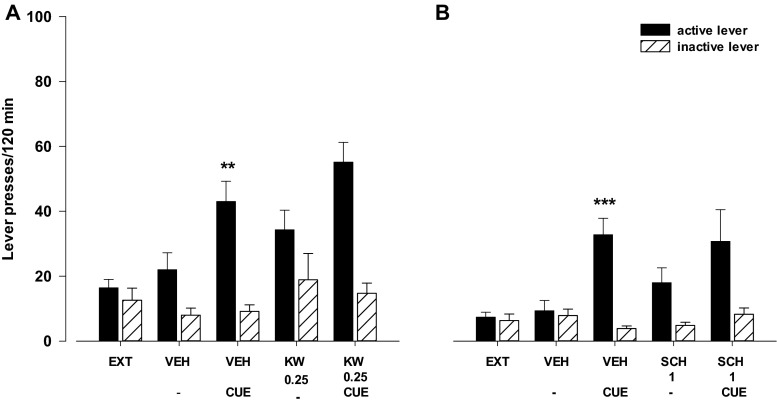
Effects of the A_2A_ receptor antagonist KW 6002 (KW; **a**) and SCH 58261 (SCH; **b**) on cue-induced reinstatement (light and tone previously associated with food self-administration) in rats extinguished from food self-administration. The number of active and inactive lever presses is shown as mean (±SEM) for seven to eight rats/group. ^**^ *p* < 0.01; ^***^ *p* < 0.001 vs. vehicle (VEH) (Newman-Keuls test)

The three-way ANOVA for pretreatment × treatment × lever interaction did not indicate a significant effect for KW 6002, 0.25 mg/kg (*F*_1.56_ = 0.29, *p* = 0.59) or SCH 58261, 1 mg/kg (*F*_1.56_ = 2.19, *p* = 0.15) on the cue induced reinstatement (Fig. 9a, b). However, the two-way ANOVA for treatment × lever interaction showed a significant effect of the cue (*F*_1.28_ = 5.23, *p* < 0.05; Fig. 9a, and *F*_1.28_ = 18.66, *p* < 0.001; Fig. 9b).

## Discussion

The current results on the actions of the A_2A_ receptor agonist CGS 21680 and the A_2A_ receptor antagonists KW 6002 and SCH 58261 on cocaine seeking give strong support to the view that A_2A_ receptor mechanisms in the rat brain block reinstatement of cocaine seeking evoked by cocaine priming or the drug-associated cue. The inhibitory actions of CGS 21680 towards cocaine- or quinpirole-induced reinstatement of seeking behaviors in rats were in line with previous studies using ip or intra-accumbal A_2A_ receptor agonist infusions (Bachtell and Self 2009; O’Neill et al. 2012). The mechanism can involve a counteraction of the D_2_ receptor- mediated cocaine seeking. In agreement with this hypothesis, CGS 21680 was four times more powerful in counteracting the D_2_-like receptor agonist quinpirole-induced relapse versus cocaine-induced relapse. Of note, CGS 21680—in a similar dose that brought down quinpirole-evoked cocaine seeking—was also a very effective blocker of the cue-induced relapse.

Similarly to cocaine seeking, food (sweetened milk) seeking appears to be under an inhibitory A_2A_ receptor control as CGS 21680 with a similar potency as observed in the above studies on cocaine seeking reduced reinstatement of active lever presses associated with food reward.

It should be underlined that CGS 21680 (0.3–1 mg/kg, but not 0.1 mg/kg) elevated reward thresholds in rats (Baldo et al. 1999), while in the current paper, the lower doses of CGS 21690 (0.05–0.1 mg/kg) effectively reduced reinstatement of seeking behavior for cocaine and food. Therefore, the inhibitory effects of the A_2A_ receptor agonist are not likely explained by an anhedonic state produced by this drug but may involve restoration of inhibitory control inter alia from the prefrontal cortex (see Chen et al. 2013). Other studies demonstrated reduced effort-related food choice behavior (Font et al. 2008) and binge-related eating disorders (Micioni di Bonaventura et al. 2012) with activation of A_2A_ receptors localized to the nucleus accumbens, which strongly support this molecular target as the inhibitory mediator of food intake. On the other hand, local injection of CGS 21680 into the nucleus accumbens had no effect on sucrose seeking in rats trained to self-administer sucrose pellets (O’Neill et al. 2012). These findings may implicate presence of distinct types of ventral striato-pallidal GABA neurons (see Carelli et al. 2000) which express or do not express A_2A_ receptors, and thus differently control food intake.

It should be noticed that CGS 21680 reduced the number of active (but not inactive) lever pressing, suggesting that the behavioral inhibition in the above studies was likely not due to nonspecific sedation or locomotor impairment. In fact, CGS 21680 at doses of 0.05–0.2 mg/kg does not inhibit locomotor activity, while at a dose of 4 mg/kg its inhibitory actions were short-lasting (up to 15 min after drug injection) (Wydra et al. 2014).

The impact of A_2A_ receptor antagonists on cocaine-seeking behavior was studied with two types of antagonists, the xanthine derivative KW 6002 and the non-xanthine derivative SCH 58261 (Filip et al. 2012). They both display a similar potency at A_2A_ receptors; however, KW 6002—when compared with SCH 58261—appears to have a somewhat higher affinity for A_2A_ protomers in A_2A_-D_2_ heteromers versus A_2A_ protomers in A_1_-A_2A_ heteromers tested in the same cell line (Orru et al. 2011). It is unknown, if this small difference also exists in the brain. KW 6002 and SCH 58261 at a dose of 0.25 and 2 mg/kg, respectively, significantly evoked cocaine-seeking behavior by increasing active lever pressing with no activity on the inactive lever. The latter finding demonstrates that A_2A_ receptor antagonist-induced seeking was not caused by increases in motor activity. Such a behavioral selectivity was lost with a higher dose of KW 6002 (0.5 mg/kg) and the drug-induced general motor enhancement is a factor responsible for the KW 6002 enhancement of the number of active lever presses at this high dose.

It is of substantial interest that lower doses of KW 6002 (0.0625 mg/kg) could also induce cocaine seeking if combined with a subthreshold dose of cocaine (0.25 mg/kg). The low dose 0.125 mg/kg of KW6002 also markedly increased the cue-induced cocaine seeking which seems to exclude a pharmacokinetic mechanism being involved in the cocaine-KW 6002 interaction in this experiment. It was unexpected that low doses of another selective and potent A_2A_ receptor antagonist SCH 58261 (0.5 mg/kg) induced neither cocaine seeking after the cocaine subthreshold dose nor did it significantly enhance the cue-induced cocaine seeking. The mechanism for this difference between the two A_2A_ receptor antagonists is unknown. However, there are indications that different A_2A_ receptor antagonists (KW 6002 and ZM 241385) can bind to different residues of the orthosteric binding site of A_2A_ receptors which can result in differential conformational dynamics of these receptor sites (Pang et al. 2013). It is therefore possible that the conformational rearrangement produced by KW 6002 is more effective than SCH 58261 to antagonize A_2A_ receptor signaling. As a result, D_2_ signaling using low doses of A_2A_ receptor antagonists is only sufficiently increased with KW 6002 which is associated with relapse or elevation in cocaine seeking.

With regard to food intake, both KW 6002 and SCH 58261 used in similar doses to enhance cocaine seeking were also able to induce food-reinforced instrumental responding. An interesting difference towards cocaine seeking was that KW 6002 and SCH 58261 did not enhance cue-induced food seeking for unknown reasons. One explanation may be that the subsets of striato-pallidal GABA neurons involved in cue-induced food seeking do not express A_2A_ receptors in contrast to the subsets involved in cue-induced cocaine seeking. Since the inactive lever presses during reinstatement tests were not significantly increased after A_2A_ receptor antagonists, as demonstrated in the current study, the possible enhancing effects of KW 6002 and SCH 58261 can be disregarded on brain motor function in animals with a history of cocaine self-administration.

A likely mechanism by which A_2A_ receptors control drug-seeking behavior depends on a basal activity of A_2A_ receptors produced by extracellular adenosine which operates via volume transmission (Fuxe et al. 2010). Various studies have revealed that glutamatergic transmission in different pathways from the prefrontal cortex to the nucleus accumbens can mediate or inhibit reinstatement of drug-seeking for cocaine or cue (Chen et al. 2013; Cornish and Kalivas 2000; Di Ciano et al. 2001; Knackstedt and Kalivas 2009; Kravitz et al. 2012; Lobo et al. 2010). It has been demonstrated with optogenetic tools that activation of the D_1_ receptor positive medium spiny neurons of the nucleus accumbens will enhance cocaine reward while increased firing of the D_2_ receptor positive medium spiny neurons within this region will suppress cocaine reward by mediating punishment (Lobo et al. 2010; Kravitz et al. 2012). Since most of A_2A_ receptors are located in the latter pathway representing the ventral striato-pallidal GABA neurons, our hypothesis is that the A_2A_ receptor agonist inhibits cocaine- and food-seeking behavior by targeting the A_2A_ receptors in the accumbens GABA neurons projecting to the ventral pallidum which is postulated to increase their firing rate.

A_2A_-D_2_ heteroreceptor complexes were discovered in cellular models and in the brain using FRET-BRET methodologies and in situ Proximity Ligation Assays (Fuxe et al. 2008; Trifilieff et al. 2011; Borroto-Escuela et al. 2013a, b). In membrane preparations from cell lines and brain regions, A_2A_ receptor agonists reduced the affinity and Gi/o coupling of D_2_ receptors likely taking place in A_2A_-D_2_ heteroreceptor complexes (Yang et al. 1995; Dasgupta et al. 1996; Fuxe et al. 2008, 2010; Borroto-Escuela et al. 2010a). It is therefore speculated that the molecular mechanism underlying the actions of the A_2A_ receptor ligands on cocaine and food seeking can involve antagonistic A_2A_-D_2_ receptor-receptor interactions at the plasma membrane level in heteroreceptor complexes and/or at the cytoplasmatic level in their signaling cascades in the striato-pallidal GABA neurons (Filip et al. 2012; Fuxe et al. 2010, 2014). Thus, it is possible that cocaine seeking may be counteracted to a substantial degree through the A_2A_ receptor agonist activation of the A_2A_ protomer reducing the D_2_ protomer signaling of the A_2A_-D_2_ heteroreceptor complex. In support of this proposal, evidence is presented that the D_2_-like antagonist raclopride counteracted the cocaine-, quinpirole-, or cue-induced reinstatement of cocaine-seeking behavior.

After treatment with A_2A_ receptor antagonists, it is instead speculated that the D_2_ receptor protomer signaling is set free from the restraining impact of the A_2A_ protomer in the A_2A_-D_2_ heteroreceptor complex which can help elicit the cocaine reinstatement. Based on these findings, it is postulated that in the striatum (including the nucleus acumbens) an endogenous adenosine tone keeps A_2A_ receptors activated and induces a brake on the D_2_ receptor protomer signaling in the A_2A_-D_2_ heteroreceptor complex in the ventral striato-pallidal GABA neurons. In view of the well-established key role of D_2_ receptors in cocaine addiction (Koob 2013), removal of this brake by A_2A_ receptor antagonists may release cocaine-seeking behavior. However, further mechanistic studies using the A_2A_-D_2_ receptor interface interacting peptides disrupting the A_2A_-D_2_ heteroreceptor complex are necessary to support our hypothesis (see Borroto-Escuela et al. 2010a, b; Fuxe et al. 2014).

Taken together, our current pharmacological studies using one A_2A_ receptor agonist and two A_2A_ receptor antagonists give strong support to the view that brain A_2A_ receptors play a major inhibitory role in cocaine and food seeking. The A_2A_ receptor agonists/antagonists are proposed to target A_2A_ receptors in the D_2_ receptor positive ventral striato-pallidal GABA neurons which may counteract the inhibitory influence of D_2_ receptors on the activity of this pathway.
